# Impact of hyperuricemia on clinical outcomes after percutaneous coronary intervention for in-stent restenosis

**DOI:** 10.1186/s12872-018-0840-2

**Published:** 2018-06-11

**Authors:** Hyung Joon Joo, Han Saem Jeong, Hyungdon Kook, Seung Hun Lee, Jae Hyoung Park, Soon Jun Hong, Cheol Woong Yu, Do-Sum Lim

**Affiliations:** Department of Cardiology, Cardiovascular Center, Korea University Anam Hospital, 126-1, 5ka, Anam-dong, Sungbuk-ku, Seoul, 136-705 Republic of Korea

**Keywords:** Uric acid, Major adverse event, In-stent restenosis, Percutaneous coronary intervention

## Abstract

**Background:**

There have been limited data on the impact of hyperuricemia on long-term clinical outcomes after percutaneous coronary intervention (PCI) for in-stent restenosis (ISR).

**Methods:**

From January 2009 to July 2015, 317 patients who underwent repeat PCI for ISR were divided into two groups: patients with normal serum uric acid (UA) levels (normal UA group) and patients with higher serum UA levels (higher UA group). The higher UA group included patients with serum UA levels > 6.8 mg/dL or patients who were taking anti-hyperuricemic medication.

**Results:**

During a median follow-up period of 1088 days, the cumulative incidence rates of major adverse event (MAE), including a composite of all-cause death, non-fatal myocardial infarction, and any revascularization, were similar between the two groups (higher UA 36.4% vs. normal UA 29.9%, *p* = 0.389, log-rank *p* = 0.367). Follow-up angiographic data showed similar outcomes of late lumen loss (0.8 ± 0.9 mm vs. 0.8 ± 1.1 mm, *p* = 0.895) and binary restenosis rate (28.1% vs. 34.7%, *p* = 0.622). Multivariate Cox regression analysis indicated higher levels of low-density lipoprotein cholesterol (hazard ratio [HR] 1.011, 95% confidence interval [CI] 1.003–1.019, *p* = 0.006) and lower left ventricular ejection fraction (HR 0.972, 95% CI 0.948–0.996, *p* = 0.022), but not UA levels, to be the independent risk predictors of MAE.

**Conclusion:**

Hyperuricemia is not associated with poor clinical outcomes after repeat PCI for ISR lesions.

**Electronic supplementary material:**

The online version of this article (10.1186/s12872-018-0840-2) contains supplementary material, which is available to authorized users.

## Background

Drug-eluting stent (DES) implantation has remarkably decreased the in-stent restenosis (ISR) rate compared with bare metal stent (BMS) implantation [1]. Long-term follow-ups of previous large clinical trials on first-generation DES showed an annual ISR rate of approximately 6–8% [2–4]. Many clinical studies reflecting real-world situations, such as complex lesion interventions and high-risk patient populations, reported higher rates of ISR [5, 6]. Although the appropriate treatment for ISR lesions, especially after DES implantation, still remains debatable, repeat percutaneous coronary intervention (PCI) for ISR lesions is becoming more frequently used, and an overall increase of ISR can be expected in the coming years. In addition, recent studies showed an extremely high rate of ISR (between 20 and 40%) at the 6–9 months angiographic follow-up after repeat PCI [7, 8].

Many clinical, lesional, and procedural risk factors for ISR have been reported, including diabetes mellitus, chronic kidney disease, complex lesion, balloon injury, and stent underexpansion [9–12]. Pathophysiologically, ISR is considered to be an intrinsic cellular and biological response after stent implantation. Inflammation has been considered to be involved in this process. A recent study showed that high-sensitivity C-reactive protein (hsCRP) was associated with ISR and poor clinical outcomes after DES implantation [13]. Other studies also suggested different circulating inflammatory biomarkers as risk predictors of ISR [14–16]. However, prognostic biomarkers after repeat PCI for ISR have not been well studied.

Uric acid (UA) is the main metabolite of purines in the human body [17]. A hyperuricemic state could inhibit endothelial nitric oxide synthesis, promote vascular smooth muscle cell proliferation, induce microvascular injury, and trigger metabolic dysregulation [18]. Therefore, hyperuricemia is associated with other cardiovascular risk factors such as hypertension, diabetes mellitus, and chronic kidney disease [19, 20]. Previous studies have shown that hyperuricemia is a significant risk factor for cardiovascular disease and mortality in the general population [21]. In addition, it was reported as an independent risk predictor of ISR after BMS implantation [22]. It was associated with poor clinical outcomes after DES implantation [23]. The aim of this study was to determine whether a high serum UA level is associated with poor clinical outcomes in patients undergoing repeat PCI for ISR lesions.

## Methods

### Study design

We screened all consecutive patients who underwent PCI at Korea University Anam Hospital between January 2009 and July 2015. Among them, 353 patients had ISR lesions (> 50% restenosis in the stent or within 5 mm of the stent edges) and underwent repeat PCI for ISR lesions. Thirty-six patients had missing data on serum UA level and were excluded. Finally, 317 patients (328 lesions) were analyzed in this study. Clinical events were monitored until June 2016 through medical record reviews and telephone calls. The present study was approved by the hospital’s institutional review board (IRB no. AN16238-002) and performed in accordance with the Declaration of Helsinki. The need for written informed consent was waived owing to the retrospective nature of the study.

### Definitions

A serum UA level of > 6.8 mg/dL was defined as hyperuricemia for both sexes [24]. This cutoff is the limit of urate solubility in the serum, and supersaturation of urate in extracellular fluid has been known to predispose a person to various pathologic conditions, including gout and cardiovascular diseases. Thus, the higher UA group included patients with hyperuricemia (serum UA level ≥6.8 mg/dL) or patients treated with anti-hyperuricemic agents such as allopurinol and febuxostat.

The primary end point, major adverse event (MAE), was defined as a composite of all-cause death; non-fatal myocardial infarction; any revascularization, including target-vessel revascularization (TVR) and non-TVR; and coronary artery bypass graft surgery. Myocardial infarction was defined as present when patients had elevated cardiac enzymes with compatible symptoms or electrocardiographic findings. Stent thrombosis was defined as definite stent thrombosis based on Academic Research Consortium Criteria [25].

### Procedures

Interventional procedures were performed according the standard clinical guidelines. Interventional strategies, including drug-coated balloon (DCB) angioplasty, DES implantation, and use of adjunctive devices and pharmacotherapy, were decided according to the operators’ discretion. Balloon pre-dilatation was performed for all ISR lesions. The first-generation DES included CYPHER® (Cordis, Johnson & Johnson, Miami Lake, FL, USA) and TAXUS™ (Boston Scientific Corp., Marlborough, MA, USA). The second-generation DES included XIENCE™ series (Abbott Vascular Devices, Temecula, CA, USA) and Endeavor® series (Medtronic Cardiovascular, Santa Rosa, CA, USA). The third-generation DES included BioMatrix (Biosensors, Singapore, Singapore) and Nobori (Terumo Corporation, Tokyo, Japan). The DCB (SeQent® Please balloon catheter; B.Braun, Melsungen, Germany) became available and was used from July 2010.

### Laboratory measurements

Laboratory profiles, including lipid panel, creatinine, glucose, hsCRP, and UA levels, were obtained within 4 weeks before the index PCI or at the index admission date. Serum UA level was measured by using an enzymatic method with an automatic biochemistry analyzer (Beckman Coulter AU 5800; Beckman Coulter Inc., Brea, CA, USA). Creatinine clearance was calculated using the Cockcroft and Gault formula [26].

### Angiographic analysis

Three radiologic technologists blinded to the patients’ treatment performed analyses with a quantitative coronary angiographic system (CASS system; Pie Medical Instruments, Maastricht, the Netherlands). By using the guiding catheter for magnification-calibration, the diameter of the reference vessel, minimal luminal diameter, and percent diameter stenosis were measured from diastolic frames in a single, matched view showing the smallest minimal luminal diameter. ISR lesions were classified according to the Mehran classification [27]. Multifocal, diffuse, proliferative, and occlusive ISR lesions were classified as non-focal-type restenosis lesions. Acute gain was calculated as the increase in minimal lumen diameter of the treated lesion immediately after the index procedure compared with that before the procedure. Late lumen loss was defined as a decrease in minimal lumen diameter of the treated lesion at the follow-up coronary angiography compared with that immediately after the index procedure. All quantitative angiographic measurements were obtained before and after PCI, and at the follow-up coronary angiography.

### Statistics

Categorical variables are reported as count (percentage), and continuous variables are reported as mean ± standard deviation. To compare the baseline clinical characteristics, angiographic features, procedural details, and the cumulative incidence of clinical events between the higher UA and normal UA groups, the chi-square test for categorical variables and Student’s *t* test (or Wilcox test) for continuous variables were performed. Kaplan–Meier survival curves with a log-rank test were generated to compare the long-term incidence of MAE between the two groups. In order to identify the risk predictors of MAE, the multivariate Cox proportional hazard model was used to evaluate the possible contributing factors. The following variables were included in the Cox regression model: age, sex, body mass index, current smoking, hypertension, diabetes mellitus, acute myocardial infarction at the index PCI, low-density lipoprotein (LDL)-cholesterol level, triglyceride level, UA level, creatinine clearance, left ventricular ejection fraction (LVEF), previous first-generation DES implantation, multivessel involvement, chronic total occlusion lesion, ISR type (III, IV), and PCI type (DES or DCB). Hazard ratios with 95% confidence intervals and *p*-values were reported. All tests were two-tailed, and p-values < 0.05 were considered statistically significant. All statistical analyses were performed using SPSS software (v20; IBM SPSS Corp., Armonk, NY, USA).

## Results

The baseline characteristics of 317 patients who underwent PCI for ISR lesions are presented in Table 1. Eighteen patients (27.3%) already treated with anti-hyperuricemic agents before UA measurement were categorized into the higher UA group. No additional patient was started on anti-hyperuricemic agents during follow-up. Among the total 317 patients, 285 had follow-up data on serum UA levels (Additional File 1:Table S1). The normal UA group showed increased UA levels at the follow-up measurement (4.8 ± 1.1 mg/dL vs. 5.2 ± 1.3 mg/dL, *p* = 0.002). There was no significant difference between the baseline and follow-up UA levels in the higher UA group (7.3 ± 1.5 mg/dL vs. 6.7 ± 2.0 mg/dL, *p* = 0.092). The significant difference in serum UA levels between the normal UA group and the higher UA group was maintained at the follow-up measurement (5.2 ± 1.3 mg/dL vs. 6.7 ± 2.0 mg/dL, *p* < 0.001).

**Table 1:** Baseline clinical characteristics

	Normal UA (*n* = 251)	Higher UA (*n* = 66)	*p*-value
Age (year)	64.6 ± 9.9	65.3 ± 10.3	0.609
Men, *n* (%)	182 (72.5)	57 (86.4)	0.030
Body mass index	24.8 ± 3.0	25.6 ± 2.9	0.056
Current smoker, *n* (%)	61 (24.3)	16 (24.2)	1.000
Hypertension, *n* (%)	175 (69.7)	53 (80.3)	0.122
Diabetes, *n* (%)	91 (36.3)	25 (37.9)	0.920
Prior MI, *n* (%)	69 (27.5)	23 (34.8)	0.308
Diagnosis at the index PCI			
SA/UA, *n* (%) NSTEMI/STEMI, *n* (%)	210 (83.7) 41 (16.3)	54 (81.8) 12 (18.2)	0.863
Laboratory findings			
Uric acid (mg/dL)	4.9 ± 1.1	7.4 ± 1.6	<.001
Total cholesterol (mg/dL)	150.1 ± 37.3	148.8 ± 43.8	0.817
LDL-C (mg/dL)	85.9 ± 29.5	90.6 ± 36.4	0.345
HDL-C (mg/dL)	43.6 ± 11.0	42.0 ± 12.7	0.333
Triglyceride (mg/dL)	127.5 ± 75.6	147.2 ± 78.1	0.064
Glucose (mg/dL)	122.5 ± 42.0	121.8 ± 42.9	0.900
Creatinine Clearance (mL/min)	72.3 ± 22.5	63.3 ± 26.2	0.006
hsCRP (mg/L)	4.3 ± 10.6	5.8 ± 12.9	0.407
LVEF (%)	56.2 ± 7.9	53.5 ± 11.4	0.095
Medications			
DAPT	248 (98.8)	64 (97.0)	0.610
Anti-hyperuricemic agent	–	18 (27.3)	–

Data were presented as *n* (%) or mean ± SD. DES, drug-eluting stent; DCB, drug-coated balloon; MI, myocardial infarction; PCI, percutaneous coronary intervention; SA, stable angina; UA, unstable angina; NSTEMI, non-ST segment elevation myocardial infarction; STEMI, ST segment elevation myocardial infarction; LDL-C, low density lipoprotein cholesterol; HDL-C, high density lipoprotein cholesterol; hsCRP, high sensitivity C-reactive protein; LVEF, left ventricular ejection fraction; DAPT, dual anti-platelet treatment

The serum UA level was 7.4 ± 1.6 mg/dL in the higher UA group and 4.9 ± 1.1 mg/dL in the normal UA group (p < 0.001). The higher UA group had more men (86.4% vs. 72.5%, *p* = 0.03) and lower creatinine clearance (63.3 ± 26.2 mL/min vs. 72.3 ± 22.5 mL/min, *p* = 0.006). The higher UA group showed a trend of higher body mass index than the normal UA group (25.6 ± 2.9 vs. 24.8 ± 3.0, *p* = 0.056).

The significant PCI characteristics of 328 ISR lesions are shown in Table 2. The diffuse type of ISR (II, III, IV) was more frequent in the higher UA group than in the normal UA group (53.5% vs. 37.0%, *p* = 0.017; Additional File 1: Table S2,). The higher UA group showed a longer interval between the previous PCI and the index PCI (1669 days vs. 990 days, *p* = 0.045). The stent diameter was statistically larger in the higher UA group than in the normal UA group (3.0 ± 0.5 mm vs. 2.9 ± 0.4 mm, p = 0.01). Qualitative comparative analysis data also showed a trend of larger target lesion reference vessel diameter in the higher UA group than in the normal UA group (3.0 ± 0.5 mm vs. 2.9 ± 0.4 mm, *p* = 0.055; Table 3). Angiographic follow-up was performed in 150 (45.7%) lesions. There were no significant differences in late lumen loss and binary restenosis rate between the two groups.

**Table 2:** Angiographic features and procedural details

	Normal UA (*n* = 257)	Higher UA (*n* = 71)	*p*-value
Previous PCI characteristics			
Stent type			
BMS, *n* (%)	21 (8.3)	5 (7.5)	
1st generation DES, *n* (%)	88 (34.9)	35 (52.2)	0.075
2nd generation DES, *n* (%)	108 (42.9)	21 (31.3)	
3rd generation DES, *n* (%)	35 (13.9)	6 (9.0)	
Stent diameter (mm)	2.9 ± 0.3	2.9 ± 0.3	0.900
Stent length (mm)	22.7 ± 7.2	22.0 ± 7.5	0.504
Median duration between previous PCI to the index procedure (day)	990	1669	0.045
Lesion characteristics at the index PCI			
Target vessel, *n* (%)			
LAD	166 (64.6)	49 (69.0)	0.478
LCX	40 (15.6)	7 (9.9)	
RCA	51 (19.8)	15 (21.1)	
Multivessel involvement, *n* (%)	166 (64.6)	53 (74.6)	0.147
CTO, *n* (%)	17 (6.6)	7 (9.9)	0.502
ISR type (II, III, IV), *n* (%)	95 (37.0)	38 (53.5)	0.017
Procedures of the index PCI			
PCI type			
DES	174 (67.7)	52 (73.2)	0.455
DCB	83 (32.3)	19 (26.8)	
DES type			
1st generation DES, *n* (%)	17 (9.8)	7 (13.5)	0.354
2nd generation DES, *n* (%)	116 (66.7)	29 (55.8)	
3rd generation DES, *n* (%)	41 (23.6)	16 (30.8)	
DES diameter (mm)	2.9 ± 0.4	3.0 ± 0.5	0.010
DES length (mm)	23.1 ± 11.5	23.8 ± 10.3	0.685
DCB diameter (mm)	2.8 ± 0.3	2.9 ± 0.3	0.344
DCB length (mm)	20.2 ± 5.2	20.0 ± 5.1	0.875

Data were presented as *n* (%) or mean ± SD. PCI, percutaneous coronary intervention; BMS, bare metal stent; DES, drug-eluting stent; LAD, left anterior descending artery; LCX, left circumflex artery; RCA right coronary artery; CTO, chronic total occlusion; ISR, in-stent restenosis; DCB, drug-coated balloon

**Table 3:** Quantitative coronary angiography analysis

	Normal UA	Higher UA	*p*-value
Index PCI			
*n*	257	71	
Pre-procedural RVD (mm)	2.9 ± 0.4	3.0 ± 0.5	0.055
Pre-procedural MLD (mm)	0.6 ± 0.4	0.6 ± 0.4	0.396
Pre-procedural DS (mm)	79.0 ± 12.6	78.5 ± 12.4	0.749
Pre-procedural lesion length (mm)	20.1 ± 11.0	20.2 ± 10.2	0.959
Post-procedural MLD (mm)	2.7 ± 0.4	2.8 ± 0.6	0.105
Post-procedural DS (%)	8.6 ± 6.8	8.5 ± 11.9	0.925
Acute gain (mm)	2.1 ± 0.5	2.2 ± 0.6	0.293
Follow-up CAG			
*n*	118	32	
Median follow-up period (day)	462	527	0.559
Target lesion RVD (mm)	2.9 ± 0.5	2.9 ± 0.5	0.888
Target lesion MLD (mm)	1.9 ± 1.1	2.0 ± 1.1	0.590
Target lesion DS (%)	36.2 ± 34.2	33.4 ± 33.0	0.686
Late lumen loss (mm)	0.8 ± 1.1	0.8 ± 0.9	0.895
Binary restenosis, *n* (%)	41 (34.7)	9 (28.1)	0.622

Data were presented as *n* (%) or mean ± SD. PCI, percutaneous coronary intervention; UA, uric acid; CAG, coronary angiography; RVD, reference vessel diameter; MLD, minimal lumen diameter; DS, diameter stenosis

**Fig. 1:**
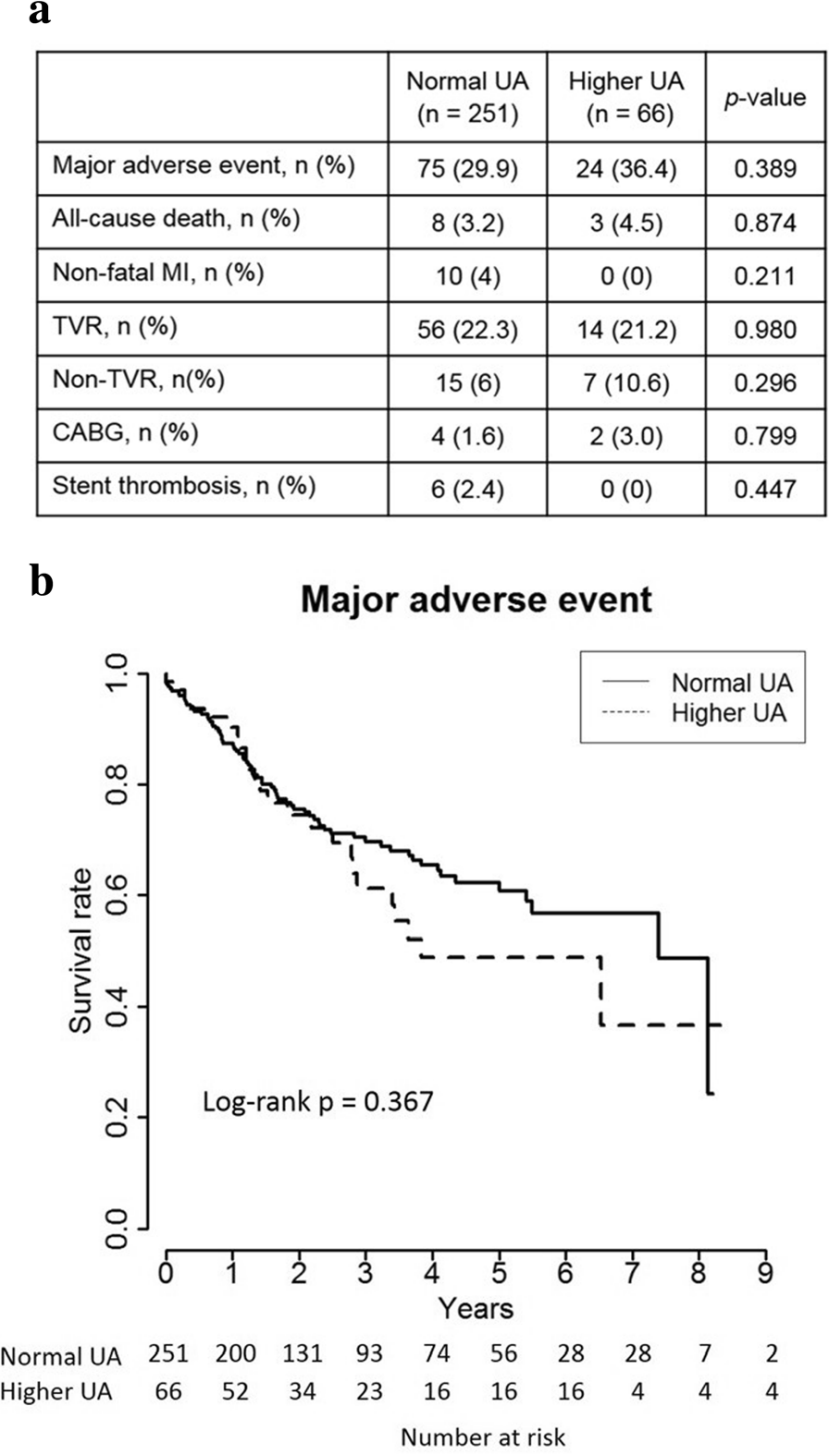
Clinical outcome. **a** Cumulative incidence of clinical events. **b** Kaplan–Meier curve for major adverse event. Data are presented as n (%). MI, myocardial infarction; TVR, target vessel revascularization; CABG, coronary artery bypass graft. Major adverse event was defined as a composite event of all-cause death, non-fatal myocardial infarction, and any revascularization, including TVR, non-TVR, and CABG

During the follow-up period (median: 748 days for the total population, 676 days for the higher UA group, and 755 days for the normal UA group; *p* = 702), the cumulative incidence rates of MAE were similar between the two groups (36.4% in the higher UA group vs. 29.9% in the normal UA group, *p* = 0.389; Fig. 1a). There were also no significant differences in other clinical events between the two groups even at different time points (Additional File 1: Table S3). Kaplan–Meier analysis indicated that the long-term incidences of MAE were similar between the two groups (log-rank test, *p* = 0.367; Fig. 1b).

Univariate Cox regression analysis suggested that serum UA level was unable to predict MAE (hazard ratio 1.110, 95% confidence interval 0.980–1.257, *p* = 0.100). Multivariate Cox regression analysis revealed that high LDL-cholesterol level and low LVEF were independent predictors of MAE (Table 4).

**Table 4:** Cox-proportional hazard models for major adverse event

	HR	95% CI	*p*-value
Univariate			
Age	1.000	0.981 – 1.019	0.992
Men	1.015	0.654 – 1.577	0.947
Body mass index	0.971	0.909 – 1.039	0.401
Current smoking	1.587	1.067 – 2.359	0.022
Hypertension	1.435	0.917 – 2.246	0.114
Diabetes mellitus	0.929	0.629 – 1.372	0.712
NSTEMI/STEMI at index PCI	1.170	0.727 – 1.884	0.518
LDL-C	1.009	1.002 – 1.015	0.008
Triglyceride	1.001	0.999 – 1.004	0.312
Uric acid	1.110	0.980 – 1.257	0.100
Creatinine clearance	0.997	0.989 – 1.006	0.494
LVEF	0.976	0.955 – 0.998	0.031
Previous 1st generation DES	1.204	0.824 – 1.758	0.338
Multivessel involvement	1.225	0.819 – 1.833	0.324
CTO lesion	0.784	0.364 – 1.689	0.534
ISR type (II, III, IV)	0.951	0.647 – 1.400	0.800
DCB (vs DES)	1.487	0.974 – 2.271	0.066
Multivariate			
Current smoking	1.521	0.957 - 2.416	0.076
Hypertension	1.469	0.870 - 2.481	0.150
LDL-C	1.011	1.003 - 1.019	0.006
LVEF	0.972	0.948 - 0.996	0.022
DCB (vs DES)	1.475	0.912 - 2.386	0.113

HR, hazard ratio; 95% CI, 95% confidence interval; NSTEMI, non-ST segment elevation myocardial infarction; STEMI, ST segment elevation myocardial infarction; LDL-C, low density lipoprotein cholesterol; LVEF, left ventricular ejection fraction; DES, drug-eluting stent; CTO, chronic total occlusion; ISR, in-stent restenosis; DCB, drug-coated balloon

## Discussion

The present study is the first to investigate the association between serum UA level and cardiovascular prognosis, especially in patients who underwent repeat PCI for ISR lesions. Hyperuricemia was present in 20.8% of patients with ISR lesions. The diffuse type of ISR was more frequent in patients with hyperuricemia. However, hyperuricemia was not associated with the incidence of MAE and angiographic ISR after re-intervention. Interestingly, high LDL-cholesterol level and low LVEF were associated with poor clinical outcomes.

### Definition of hyperuricemia

Many studies have reported that hyperuricemia is associated with cardiovascular disease. A recent meta-analysis including 29 prospective cohort studies also showed that hyperuricemia is an independent risk factor for cardiovascular morbidity and mortality [28]. Biologically, UA exerts pro-oxidant or nitric-oxide-reducing effects depending on its concentration and chemical microenvironment [29]. When the urate concentration exceeds 6 mg/dL, the risk of urate crystal formation and precipitation increases. Therefore, hyperuricemia is generally defined as a serum UA level of > 6.8 mg/dL [30]. The present study adopted this cutoff value. However, the optimal threshold for serum UA level remains debatable. Some studies used different cutoff values based on sex, considering the significant difference in reference ranges of serum UA levels between men and women. Recently, the clinically detrimental effect of serum UA seems to be evident even below its saturation limit, likely independent of urate crystal formation in cardiovascular diseases. Receiver-operating characteristic curve analysis of serum UA level for MAE in the present study showed an area under the curve of 0.544 (95% confidence interval 0.474–0.615, data not shown). In addition, when we further analyzed the clinical outcomes between two groups determined using the median UA level (5.3 mg/dL), the results also showed similar clinical outcomes between patients with lower UA level (≤5.3 mg/dL) and patients with higher UA level (> 5.3 mg/dL) (Additional File 1: Table S4 and Additional File 1: Figure S1). These data suggested that the association between serum UA level and poor clinical outcomes was very weak, and the optimal cutoff value of hyperuricemia might be obscure in those high-risk patients who underwent repeat PCI for ISR lesions.

### Clinical and angiographic characteristics of hyperuricemic patients

In the present study, patients with hyperuricemia were predominantly male and somewhat obese. Additionally, they had lower creatinine clearance and showed a trend of higher serum triglyceride levels. Interestingly, patients with hyperuricemia had a higher frequency of non-focal-type restenosis lesions than normouricemic patients. Previously, elevated serum hsCRP level was reported as a risk predictor of non-focal-type ISR after DES implantation, suggesting that inflammatory activity might contribute to aggressive restenosis [31]. In addition, old age, hypertension, diabetes mellitus, and paclitaxel-eluting stent implantation were also reported to be associated with the non-focal type of ISR [32–34]. Thus, considering that hyperuricemia is associated with elevated hsCRP level and other inflammatory markers, it could also be another possible biomarker for non-focal-type ISR. In addition, the present study showed a significant difference in the interval between previous PCI and index PCI between the low UA group and the high UA group. The high UA group took a longer time to develop ISR than the lower UA group. A previous study using an intravascular imaging modality demonstrated that neointimal hyperplasia is associated with earlier ISR, whereas neoatherosclerosis is associated with later ISR [35]. It also suggested the potential role of a high serum UA level in the development of neoatherosclerosis and ISR.

### Risk predictors for poor prognosis after repeat PCI for ISR

The present study did not show an association between hyperuricemia and clinical outcomes after repeat PCI for ISR lesions. Previous stent type, stent number, bifurcation lesion, ISR type, and repeat first-generation DES implantation were suggested as risk predictors of poor prognosis [36–39]. Conventional demographic risk factors, such as diabetes mellitus, failed to reach clinical significance after repeat PCI for ISR [40]. These findings suggested that the pathologic mechanisms of recurrent ISR are rather different from those of de novo coronary atherosclerosis, and implied that lesional, technical, and mechanical factors might play important roles in recurrent ISR development after repeat PCI for ISR. A recent study even suggested DCB angioplasty as a predictor of target lesion failure in the second-generation DES era [41]. When we analyzed the impact of hyperuricemia in patients treated with DES or in patients treated with DCB separately, there were no significant differences in clinical outcomes between the low UA group and the high UA group in both the DES- and DCB-treated patients (Additional File 1: Table S5).

In addition, it was previously demonstrated that serum LDL-cholesterol level was significantly associated with the development of neoatherosclerosis, which has been studied as an important pathologic process related to poor clinical outcome after PCI in the DES era [42]. There was also a case of recurrent neoatherosclerosis after repeat PCI for ISR [43]. These data suggested that the residual risk of altered lipid metabolism should be considered after repeat PCI for ISR lesions. The present study indicated LDL-cholesterol level and LVEF as important risk predictors of MAEs (Table 4). However, the Cox proportional hazard model for TVR failed to suggest any independent risk factor from the 17 potential risk factors including age, sex, body mass index, current smoking, hypertension, diabetes mellitus, presentation of acute myocardial infarction, LDL-C, triglyceride, UA, creatinine clearance, LVEF, prior first-generation DES use, multivessel involvement, chronic total occlusion, ISR type, and PCI strategy (data not shown). The Cox proportional hazard model for non-TVR proposed LDL-C and LVEF as the independent risk factors for non-TVR in patients after repeat PCI for ISR (Additional File 1: Table S6). These results suggested that LDL-C and LVEF contribute to MAE development mainly driven by non-TVR rather than TVR. Management of lipid profile and heart failure could be emphasized as a fundamental strategy to prevent adverse clinical outcomes in patients after repeat PCI for ISR, although their association with TVR is obscure. However, the present study showed that TVR rather than non-TVR formed a majority of MAEs (70.7%, 70 of 99). Thus, although the present study failed to suggest the important risk predictor for repeat target vessel failure, further studies should resolve this issue.

### Study limitations

The present study has several limitations. First, this is a single-center, retrospective study. The study population was enrolled for a long duration and the baseline characteristics were heterogeneous. Moreover, the PCI strategy was dependent on the discretion of the operators, and a selection bias should be considered in the interpretation of our results. Second, the sample size was too small to discriminate the clinical impact of hyperuricemia, although the patients were followed-up for a long duration. Third, the present study did not analyze intravascular imaging data (intravascular ultrasound or optical coherence tomography) because of their limited usage (36.9%). Considering that mechanical and technical factors may contribute to ISR, detailed lesional information could provide an insight into the clinical relevance of hyperuricemia. Therefore, our findings should be extended and validated further by other studies.

## Conclusions

The non-focal-type ISR lesion was more frequent in patients with hyperuricemia. However, hyperuricemia was not associated with poor clinical outcomes after repeat PCI for ISR lesions. Serum LDL-cholesterol level and LVEF were independent risk predictors of poor clinical outcomes.

## Additional file

Additional file 1: **Table S1.** Changes in serum uric acid level, **Table S2.** In-stent restenosis patterns at the index procedure, **Table S3.** Cumulative incidence of clinical events between patients with lower uric acid level (≤6.8 mg/dL) and those with higher uric acid level (> 6.8 mg/dL), **Table S4.** Cumulative incidence of clinical events between patients with lower uric acid level (≤5.3 mg/dL) and those with higher uric acid level (> 5.3 mg/dL), **Table S5.** Subgroup analyses of the cumulative incidence of clinical events between the low uric acid group and the high uric acid group, **Table S6.** Cox-proportional hazard models for non-target vessel revascularization, **Figure S1.** Kaplan–Meier curve for major adverse event between patients with lower uric acid level (≤5.3 mg/dL) and those with higher uric acid level (> 5.3 mg/dL). UA, uric acid. (DOCX 134 kb)

## Abbreviations

BMS: Bare metal stent
DCB: Drug-coated balloon
DES: Drug-eluting stent
hsCRP: High-sensitivity C-reactive protein
ISR: In-stent restenosis
LDL: Low-density lipoprotein
LVEF: Left ventricular ejection fraction
MAE: Major adverse event
PCI: Percutaneous coronary intervention
TVR: Target-vessel revascularization
UA: Uric acid.
