# Supplementary material for: Impact of hyperuricemia on clinical outcomes after percutaneous coronary intervention for in-stent restenosis
Source: BMC Cardiovasc Disord. 2018 Jun 11;18:114. doi: 10.1186/s12872-018-0840-2 (PMC5996510; doi:10.1186/s12872-018-0840-2)

Additional File 1

Additional File Table S1. Changes in serum uric acid level

|  | Baseline (mg/dL) | Follow-up (mg/dL) | p-Value |
| --- | --- | --- | --- |
| Low UA (n = 225) | 4.8 ± 1.1 | 5.2 ± 1.3 | 0.002 |
| High UA (n = 60) | 7.3 ± 1.5 | 6.7 ± 2.0 | 0.092 |

UA, uric acid.

Additional File **Table 2. In-stent restenosis patterns at the index procedure**

|  | Normal UA  (n = 257) | Higher UA  (n = 71) | p-Value |
| --- | --- | --- | --- |
| Type I  Type 1a  Type 1b  Type 1c  Type 1d | 162 (63.0)  4 (1.6)  84 (32.7)  71 (27.6)  3 (1.2) | 33 (46.5)  2 (2.8)  21 (29.6)  9 (12.7)  1 (1.4) | 0.127 |
| Type II | 44 (17.1) | 16 (22.5) |  |
| Type III | 23 (8.9) | 11 (15.5) |  |
| Type IV | 28 (10.9) | 11 (15.5) |  |

Additional File **Table 3. Cumulative incidence of clinical events between patients with lower uric acid level (≤6.8 mg/dL) and those with higher uric acid level (>6.8 mg/dL)**

| Year | Clinical event | Lower UA  (n = 251) | Higher UA  (n = 66) | p-Value |
| --- | --- | --- | --- | --- |
| 1 | Major adverse event, n (%) | 29 (11.6) | 6 (9.1) | 0.728 |
|  | All-cause death, n (%) | 3 (1.2) | 1 (1.5) | 1.000 |
|  | Non-fatal MI, n (%) | 4 (1.6) | 0 | 0.680 |
|  | TVR, n (%) | 19 (7.6) | 2 (3.0) | 0.298 |
|  | Non-TVR, n (%) | 9 (3.6) | 2 (3.0) | 1.000 |
|  | CABG, n (%) | 0 | 1 (1.5) | 0.472 |
|  | Stent thrombosis, n (%) | 3 (1.2) | 0 | 0.859 |
| 3 | Major adverse event, n (%) | 62 (24.7) | 19 (28.8) | 0.604 |
|  | All-cause death, n (%) | 6 (2.4) | 3 (4.5) | 0.602 |
|  | Non-fatal MI, n (%) | 9 (3.6) | 0 | 0.253 |
|  | TVR, n (%) | 46 (18.3) | 9 (13.6) | 0.476 |
|  | Non-TVR, n (%) | 13 (5.2) | 6 (9.1) | 0.368 |
|  | CABG, n (%) | 2 (0.8) | 2 (3.0) | 0.408 |
|  | Stent thrombosis, n (%) | 5 (2.0) | 0 | 0.548 |
| 5 | Major adverse event, n (%) | 70 (27.9) | 23 (34.8) | 0.341 |
|  | All-cause death, n (%) | 7 (2.8) | 3 (4.5) | 0.741 |
|  | Non-fatal MI, n (%) | 10 (4.0) | 0 | 0.211 |
|  | TVR, n (%) | 52 (20.7) | 13 (19.7) | 0.991 |
|  | Non-TVR, n (%) | 14 (5.6) | 7 (10.6) | 0.237 |
|  | CABG, n (%) | 3 (1.2) | 2 (3.0) | 0.610 |
|  | Stent thrombosis, n (%) | 6 (2.4) | 0 | 0.447 |
| 7 | Major adverse event, n (%) | 73 (29.1) | 24 (36.4) | 0.321 |
|  | All-cause death, n (%) | 7 (2.8) | 3 (4.5) | 0.741 |
|  | Non-fatal MI, n (%) | 10 (4.0) | 0 | 0.211 |
|  | TVR, n (%) | 54 (21.5) | 14 (21.2) | 1.000 |
|  | Non-TVR, n (%) | 15 (6.0) | 7 (10.6) | 0.296 |
|  | CABG, n (%) | 4 (1.6) | 2 (3.0) | 0.799 |
|  | Stent thrombosis, n (%) | 6 (2.4) | 0 | 0.447 |
| Total | Major adverse event, n (%) | 75 (29.9) | 24 (36.4) | 0.389 |
|  | All-cause death, n (%) | 8 (3.2) | 3 (4.5) | 0.874 |
|  | Non-fatal MI, n (%) | 10 (4) | 0 | 0.211 |
|  | TVR, n (%) | 56 (22.3) | 14 (21.2) | 0.980 |
|  | Non-TVR, n (%) | 15 (6) | 7 (10.6) | 0.296 |
|  | CABG, n (%) | 4 (1.6) | 2 (3.0) | 0.799 |
|  | Stent thrombosis, n (%) | 6 (2.4) | 0 | 0.447 |

Data are presented as n (%). UA, uric acid; MI, myocardial infarction; TVR, target vessel revascularization; CABG, coronary artery bypass graft. Major adverse event was defined as a composite event of all-cause death, non-fatal myocardial infarction, and any revascularization, including TVR, non-TVR, and CABG.

Additional File **Table 4. Cumulative incidence of clinical events between patients with lower uric acid level (≤5.3 mg/dL) and those with higher uric acid level (>5.3 mg/dL)**

|  | Lower UA  (≤5.3 mg/dL, n = 160) | Higher UA  (>5.3 mg/dL, n = 157) | p-Value |
| --- | --- | --- | --- |
| Major adverse event, n (%) | 45 (28.1) | 54 (34.4) | 0.279 |
| All-cause death, n (%) | 4 (2.5) | 7 (4.5) | 0.518 |
| Non-fatal MI, n (%) | 8 (5.0) | 2 (1.3) | 0.115 |
| TVR, n (%) | 34 (21.2) | 36 (22.9) | 0.822 |
| Non-TVR, n (%) | 9 (5.6) | 13 (8.3) | 0.478 |
| CABG, n (%) | 3 (1.9) | 3 (1.9) | 1.000 |
| Stent thrombosis, n (%) | 4 (2.5) | 2 (1.3) | 0.697 |

Data are presented as n (%). UA, uric acid; MI, myocardial infarction; TVR, target vessel revascularization; CABG, coronary artery bypass graft. Major adverse event was defined as a composite event of all-cause death, non-fatal myocardial infarction, and any revascularization, including TVR, non-TVR, and CABG.

Additional File **Table 5. Subgroup analyses of the cumulative incidence of clinical events between the low uric acid group and the high uric acid group**

| Event | DES | | | DCB | | |
| --- | --- | --- | --- | --- | --- | --- |
|  | Low UA  (n = 168) | High UA  (n = 48) | p-Value | Low UA  (n = 83) | High UA  (n = 18) | p-Value |
| MAE, n (%) | 52 (31.0) | 16 (33.3) | 0.891 | 23 (27.7) | 8 (44.4) | 0.265 |
| All-cause death, n (%) | 6 (3.6) | 1 (2.1) | 0.959 | 2 (2.4) | 2 (11.1) | 0.294 |
| Non-fatal MI, n (%) | 6 (3.6) | 0 | 0.407 | 4 (4.8) | 0 | 0.777 |
| TVR, n (%) | 39 (23.2) | 10 (20.8) | 0.879 | 17 (20.5) | 4 (22.2) | 1.000 |
| Non-TVR, n (%) | 10 (6.0) | 5 (10.4) | 0.453 | 5 (6.0) | 2 (11.1) | 0.796 |
| CABG, n (%) | 2 (1.2) | 1 (2.1) | 1.000 | 2 (2.4) | 1 (5.6) | 1.000 |
| Stent thrombosis, n (%) | 6 (3.6) | 0 | 0.407 | 0 | 0 | - |

Data are presented as n (%). DES, drug-eluting stent; DCB, drug-coated balloon; UA, uric acid; MAE, major adverse event; MI, myocardial infarction; TVR, target vessel revascularization; CABG, coronary artery bypass graft. Major adverse event was defined as a composite event of all-cause death, non-fatal myocardial infarction, and any revascularization, including TVR, non-TVR, and CABG.

Additional File **Table 6. Cox-proportional hazard models for non-target vessel revascularization**

|  | HR | 95% CI | p-Value |
| --- | --- | --- | --- |
| **Univariate** | | | |
| Age | 1.025 | 0.981–1.071 | 0.273 |
| Men | 0.708 | 0.240–2.092 | 0.532 |
| Body mass index | 1.018 | 0.882–1.175 | 0.808 |
| Current smoking | 1.443 | 0.588–3.540 | 0.424 |
| Hypertension | 1.344 | 0.496–3.644 | 0.561 |
| Diabetes mellitus | 1.405 | 0.607–3.253 | 0.428 |
| NSTEMI/STEMI at index PCI | 0.784 | 0.232–2.653 | 0.696 |
| LDL-C | 1.017 | 1.005–1.028 | 0.004 |
| Triglyceride | 1.004 | 1.000–1.009 | 0.034 |
| Uric acid | 1.317 | 1.047–1.656 | 0.019 |
| Creatinine clearance | 0.995 | 0.977–1.014 | 0.626 |
| LVEF | 0.964 | 0.924–1.005 | 0.086 |
| Previous first-generation DES | 0.996 | 0.425–2.332 | 0.992 |
| Multivessel involvement | 3.463 | 1.024–11.71 | 0.046 |
| CTO lesion | 0.609 | 0.082–4.534 | 0.628 |
| ISR type (II, III, IV) | 0.697 | 0.284–1.709 | 0.430 |
| DCB (vs. DES) | 1.340 | 0.559–3.451 | 0.479 |
| **Multivariate** | | | |
| LDL-C | 1.015 | 1.000–1.029 | 0.045 |
| Triglyceride | 1.004 | 0.999–1.009 | 0.093 |
| LVEF | 0.955 | 0.914–0.998 | 0.040 |
| Multivessel involvement | 2.916 | 0.852–9.980 | 0.088 |

HR, hazard ratio; 95% CI, 95% confidence interval; NSTEMI, non-ST segment elevation myocardial infarction; STEMI, ST segment elevation myocardial infarction; LDL-C, low density lipoprotein cholesterol; LVEF, left ventricular ejection fraction; DES, drug-eluting stent; CTO, chronic total occlusion; ISR, in-stent restenosis; DCB, drug-coated balloon.

Additional File **Figure 1. Kaplan–Meier curve for major adverse event between patients with lower uric acid level (≤5.3 mg/dL) and those with higher uric acid level (>5.3 mg/dL). UA, uric acid.**


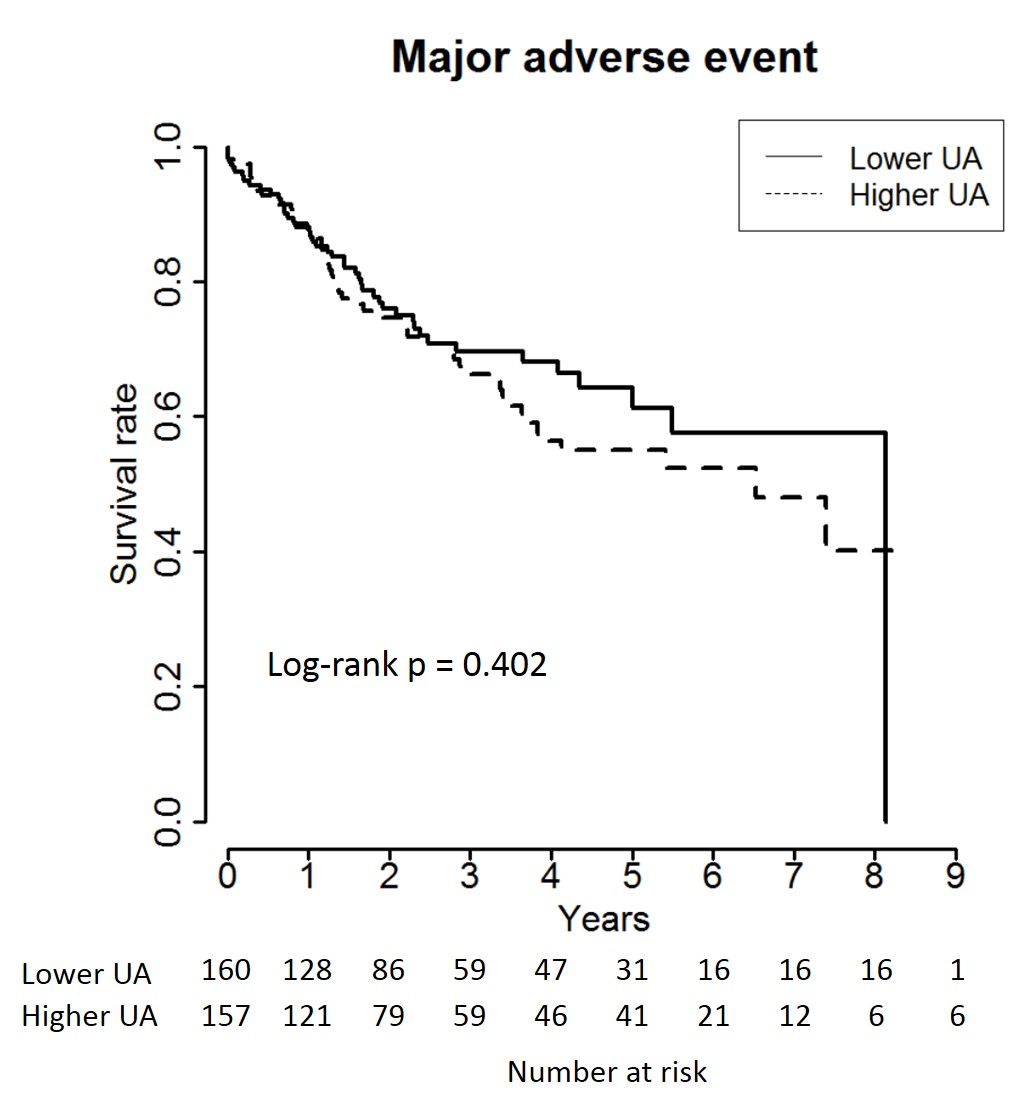

Supplement: Supplementary file 1 — Table S1. Changes in serum uric acid level, Table S2. In-stent restenosis patterns at the index procedure, Table S3. Cumulative incidence of clinical events between patients with lower uric acid level (≤6.8 mg/dL) and those with higher uric acid level (> 6.8 mg/dL), Table S4. Cumulative incidence of clinical events between patients with lower uric acid level (≤5.3 mg/dL) and those with higher uric acid level (> 5.3 mg/dL), Table S5. Subgroup analyses of the cumulative incidence of clinical events between the low uric acid group and the high uric acid group, Table S6. Cox-proportional hazard models for non-target vessel revascularization, Figure S1. Kaplan–Meier curve for major adverse event between patients with lower uric acid level (≤5.3 mg/dL) and those with higher uric acid level (> 5.3 mg/dL). UA, uric acid. (DOCX 134 kb) [file 12872_2018_840_MOESM1_ESM.docx]
